# Longitudinal association of adverse childhood experiences with cognitive function trajectories among middle-aged and older adults: group-based trajectory modeling

**DOI:** 10.3389/fpsyt.2024.1440265

**Published:** 2024-08-06

**Authors:** Xingyue He, Hui Yang

**Affiliations:** ^1^ School of Nursing, Shanxi Medical University, Taiyuan, China; ^2^ Department of Nursing, The First Hospital of Shanxi Medical University, Taiyuan, China

**Keywords:** adverse childhood experiences, cognitive function, group-based trajectories, predictors, CHARLS

## Abstract

**Introduction:**

Adverse childhood experiences (ACEs) impact cognitive function, but the relationship remains unclear. We aim to identify cognitive function trajectories and scrutinize the correlation between ACEs and cognitive function.

**Methods:**

To identify cognitive trajectories, we employed a group-based trajectory model, and influential factors were determined using multinomial unordered logistic regression analysis.

**Results:**

Three cognitive decline subgroups emerged: low-start decline, high-start stability, and mid-start decline. There is no dose-response relationship between cumulative adverse childhood experiences and cognitive function. The high-start stability group had specific residence and education traits, while sibling death affected them. The mid-start decline group was vulnerable to parental death, physical abuse, and domestic violence. The low-start decline group should consider age structure and childhood friendships.

**Conclusions:**

No dose-response association between cumulative ACEs and cognitive decline. Still, specific ACE metrics are correlated with cognitive trajectories. We can incorporate patients’ ACEs into cognitive function assessments for early risk factor identification and tailored interventions. Moreover, recognizing the influence of early-life experiences on cognitive function, we can advocate for nurturing positive family and societal environments to optimize cognitive function.

## 1 Introduction

The world’s population is growing older. Cognitive function declines with age, characterized by the clinical syndrome of dementia, which can lead to significant social and economic costs for caregivers and society (1). Therefore, identifying modifiable risk factors for cognitive decline can inform the development of interventions and strategies for healthy aging (2).

According to the life course theory, cognitive functioning in middle-aged and older adults is influenced not only by their current circumstances but also by the accumulation of life experiences (3). Adverse childhood experiences(ACEs) are related to cognitive function in middle-aged and older adults (3, 4). Specifically, ACEs represent various adverse events before the age of 17, including direct and environmental events (5, 6). Direct events may include sexual abuse, physical abuse, psychological abuse, and physical and emotional neglect, while environmental events often describe those not directly targeted at children, such as witnessing parental violence, living with someone with psychopathology or imprisonment, or experiencing parental separation or divorce (7). A study in the UK reported that nearly half of respondents, adults to 70-year-olds, reported at least one ACE, and 12% reported four or more ACEs (8). Significantly, ACEs are associated with a higher likelihood of severe neurocognitive disorders. For example, exposure to early-life adversity can increase the likelihood of developing late-onset dementia by 2.15-4.22 times (9, 10). These studies show the adverse effects of ACEs on cognitive function.

Despite ACEs profoundly impacting cognitive function, the relationship remains elusive, with inconsistencies in existing studies. Some studies indicate a dose-response relationship between ACEs and cognitive decline, with higher ACE exposure associated with greater risk (10–12). Conversely, other studies have reported that ACEs were linked to lower baseline cognitive function but did not significantly impact cognitive function decline (13). In a Japanese survey of older adults, ACEs were only associated with reduced cognitive function in those with low social capital. Therefore, there is a need to verify the relationship between ACEs and cognitive function.

Furthermore, given the dynamic and personalized nature of cognitive function, longitudinal investigations offer a suitable approach to unveil the potential role of ACEs in cognitive changes (14). Most longitudinal studies have used general linear or linear mixed models to assess the link between ACEs and cognitive function. However, these methods may have limitations in revealing distinct developmental trajectories among subgroups (13, 15, 16). The group-based trajectory model (GBTM) can estimate multiple trajectories simultaneously, and several studies have utilized group-based trajectory models to analyze cognitive trajectories (17–19). No study has used GBTM to investigate the association between ACEs and cognitive function trajectories.

Given this, we aim to address the following critical questions regarding cognitive function in middle-aged and older adults (1): What are cognitive function trajectory types’ heterogeneity and distribution characteristics? (2) Are cumulative ACEs associated with cognitive function trajectory types? (3) What are the predictors of different cognitive function trajectories?

## 2 Methods

### 2.1 Study design and sample

We used the deidentified data from the China Health and Retirement Longitudinal Study (CHARLS) cohort. The study sample was obtained by four-stage stratified sampling using the probability-proportional-to-size technique. The baseline survey of CHARLS, conducted in 2011, included 17,708 respondents from 28 provinces. Three follow-up assessments were performed in 2013, 2015, and 2018. In addition, a life history survey was conducted in 2014. The details of the CHARLS have been published elsewhere (20). The CHARLS program complied with the principles of the Declaration of Helsinki and received ethical approval from the Peking University Institutional Review Board (IRB00001052-11015) (20, 21). All participants in the CHARLS provided written informed consent.

We used data from the 2011 CHARLS baseline survey, all three follow-up assessments, and the 2014 life history survey. After excluding participants who passed away or missed follow-up assessments, the final sample comprised 1679 respondents who met the following criteria: (1) baseline age ≥ 45, (2) complete all cognitive assessments, and (3) provided ACEs information (**Supplementary Figure 1** in **Supplementary Material**).

### 2.2 Measures

#### 2.2.1 Definition of adverse childhood experiences

We captured relevant indicators of ACEs (**Supplementary Table 1** in **Supplementary Material**) (22–24), including child maltreatment, exposure to violence, parent/sibling death or disability, and parental maladjustment. Each participant’s cumulative ACEs were calculated by dichotomizing responses for each item and summing them up, thereby categorizing participants into four groups based on cumulative ACEs: 1, 2, 3, and ≥4.

#### 2.2.2 Cognitive function assessment

A total cognitive function score measured episodic memory and mental intactness (**Supplementary Table 2** in **Supplementary Material**) (25–28). The assessment of episodic memory encompasses both immediate recall and delayed recall. The assessment of mental intactness includes time orientation, overlapping pentagon drawing, and arithmetic. Consistent with prior CHARLS publications, the total cognitive function score was the sum of the episodic memory and mental intactness scores (ranging from 0 to 21, Cronbach’s α = 0.835) and higher scores indicating better cognitive functioning (25–28).

#### 2.2.3 Covariates

**Figure 1:**
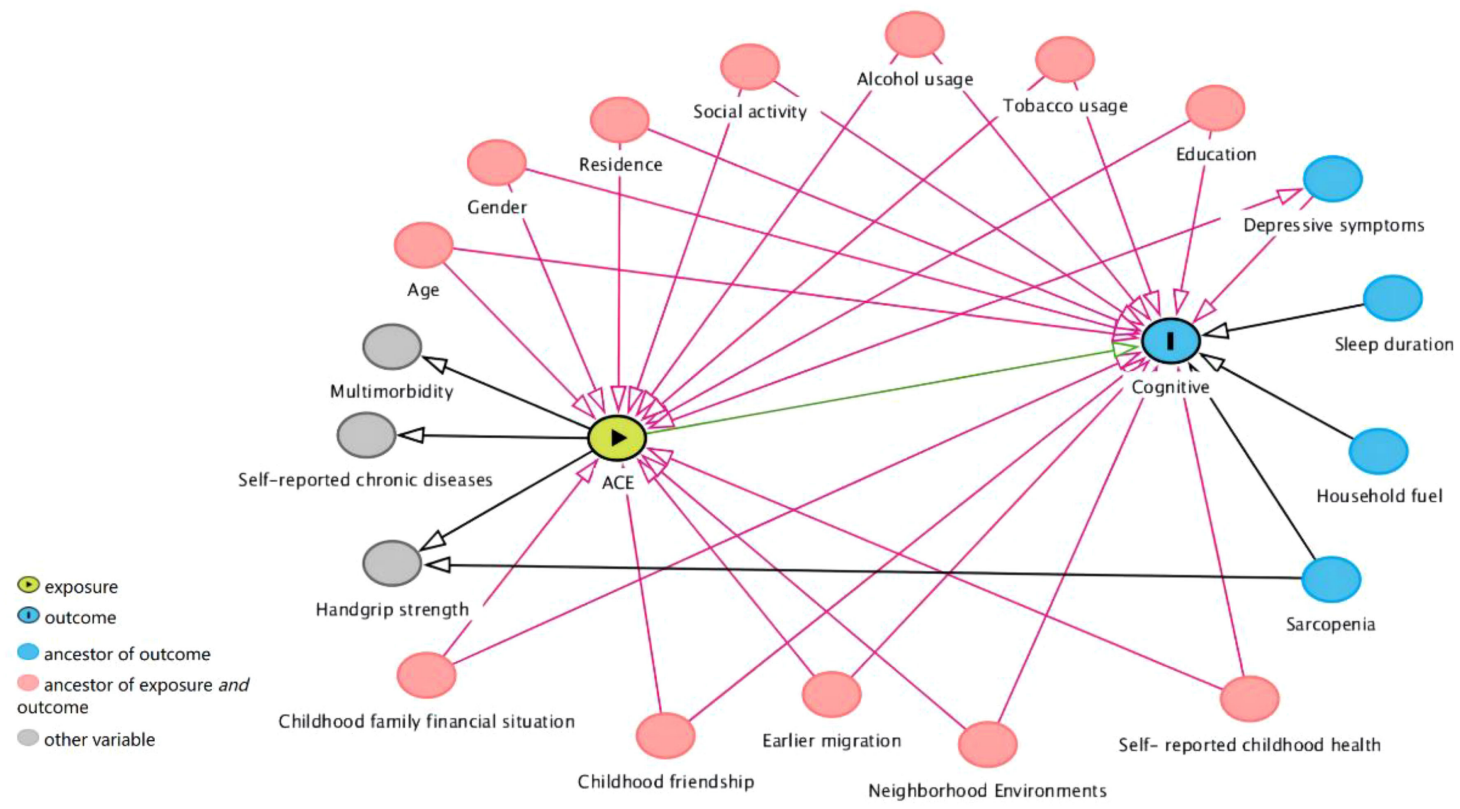
Directed acyclic graphs. Ancestor of outcome: preceding or prior nodes that influence a specific outcome in a directed acyclic graph. Ancestor of exposure and outcome: nodes that influence both the exposure and outcome within a causal relationship context in a directed acyclic graph.

We use directed acyclic graphs to identify potential confounding factors (13, 25, 29, 30) (**Figure 1**). We considered demographic variables (age, residence, education, gender), childhood-related variables (earlier migration, childhood friendship, neighborhood environments, childhood family financial situation, self-reported childhood health), and health behaviors (tobacco usage, alcohol usage, and social activity).

### 2.3 Statistical approach

Firstly, We used Group-based Trajectory Modelling (GBTM) (31) to identify groups of cognitive functions using the Stata Traj plug-in (32). Various factors were taken into account when fitting the trajectory model, including statistical measures, visual inspection of predicted trajectories, Bayesian Information Criterion (BIC, where smaller values indicate better model fit), Average Posterior Probability (AvePP; exceeding 0.7 indicates the best fit), and representation of group members with probabilities ≥5% (17). Following the model selection criteria of GBTM, we iteratively adjusted the number of groups (2-4 group models) and trajectory shapes (from cubic to linear).

Secondly, in describing the characteristics of the study sample, we treated all independent variables as categorical variables, reported them using frequencies (percentages), and utilized χ2 tests for comparisons between groups to explore whether participant characteristics differed between cognitive trajectory groups.

Finally, the identified trajectory types were used as dependent variables, and the connection between ACEs and cognitive function trajectories was analyzed using multinomial unordered logistic models. The multinomial unordered logistic model was used, with the low-start decline group designated as the reference group. Considering the influence of interactions between variables, all variables are included in the multinomial unordered logistic regression for screening analysis. The *p*-values correspond to two-tailed tests, with statistical significance at *p* < 0.05.

We reported our study using the Strengthening the Reporting of Observational Studies in Epidemiology (STROBE) reporting guideline (**Supplementary Table 3** in **Supplementary Material**).

## 3 Results

### 3.1 Sample characteristics

The baseline characteristics of each group of individuals in the cognitive function trajectory are summarized in **Table 1**. Significant differences were observed among members of the cognitive function trajectory groups in terms of gender, age, residence, social activity, education, childhood family financial situation, childhood friendships, earlier migration, neighborhood environment, and self-reported childhood health (*p*<0.05). Notably, regarding alcohol and tobacco usage, there were no notable variations within the cognitive function trajectory subgroup (*p* > 0.05).

**Table 1:** Baseline characteristics of the total sample and the sample by the different trajectory groups^a^.

Characteristic	Trajectory group				
	Total sample (N= 1,679)	Low-start decline group (N=195)	High-start stability group (N= 835)	Mid-start decline group (N=649)	*p-*value*
Gender					0.006
male	940 (55.99)	92 (47.18)	494 (59.16)	354 (54.55)	
female	739 (44.01)	103 (52.82)	341 (40.84)	295 (45.45)	
Age					<0.001
45-54	556 (33.11)	38 (19.49)	316 (37.84)	202 (31.12)	
55-64	671 (39.96)	77 (39.49)	327 (39.16)	267 (41.14)	
≥65	452 (26.92)	80 (41.03)	192 (22.99)	180 (27.73)	
Residence					<0.001
agricultual Hukou	1271 (75.70)	185 (94.87)	534 (63.95)	552 (85.05)	
non-agricultural Hukou	408 (24.30)	10 (5.13)	301 (36.05)	97 (14.95)	
Social activity					0.003
none	789 (46.99)	107 (54.87)	360 (43.11)	322 (49.61)	
yes	890 (53.01)	88 (45.13)	475 (56.89)	327 (50.39)	
Alcohol usage					0.464
none of these	1068 (63.61)	133 (68.21)	519 (62.16)	416 (64.10)	
drink more than once a month	462 (27.52)	44 (22.56)	238 (28.50)	180 (27.73)	
drink but less than once a month	149 (8.87)	18 (9.23)	78 (9.34)	53 (8.17)	
Tobacco usage					0.192
none	931 (55.45)	113 (57.95)	476 (57.01)	342 (52.70)	
yes	748 (44.55)	82 (42.05)	359 (42.99)	307 (47.30)	
Education					<0.001
no formal education	92 (5.48)	47 (24.1)	11 (1.32)	34 (5.24)	
≤middle school	1328 (79.09)	147 (75.38)	603 (72.22)	578 (89.06)	
≥high school/Vocational school	25 (15.43)	1 (0.51)	221 (26.47)	37 (5.7)	
Childhood family financial situation					0.007
financially advantaged	16 (0.95)	2 (1.03)	10 (1.20)	4 (0.62)	
financially better off	157 (9.35)	9 (4.62)	97 (11.62)	51 (7.86)	
financially equal	894 (53.25)	99 (50.77)	437 (52.34)	358 (55.16)	
financially somewhat worse off	257 (15.31)	39 (20.00)	132 (15.81)	86 (13.25)	
financially disadvantaged	355 (21.14)	46 (23.59)	159 (19.04)	150 (23.11)	
Childhood friendship					<0.001
often	1133 (67.48)	98 (50.26)	616 (73.77)	419 (64.56)	
sometimes	230 (13.70)	37 (18.97)	110 (13.17)	83 (12.79)	
not very often	147 (8.76)	23 (11.79)	53 (6.35)	71 (10.94)	
never	169 (10.07)	37 (18.97)	56 (6.71)	76 (11.71)	
Earlier migration					<0.001
no move out residence	1104 (65.75)	144 (73.85)	508 (60.84)	452 (69.65)	
move out residence	575 (34.25)	51 (26.15)	327 (39.16)	197 (30.35)	
Neighborhood environments					0.021
very close-knit	760 (45.27)	83 (42.56)	405 (48.5)	272 (41.91)	
somewhat close-knit	855 (50.92)	98 (50.26)	408 (48.86)	349 (53.78)	
not very close-knit	55 (3.28)	12 (6.15)	19 (2.28)	24 (3.70)	
not close-knit at all	9 (0.54)	2 (1.03)	3 (0.36)	4 (0.62)	
Self-reported childhood health					0.016
much healthier	277 (16.5)	25 (12.82)	141 (16.89)	111 (17.1)	
somewhat healthier	351 (20.91)	38 (19.49)	204 (24.43)	109 (16.8)	
about average	845 (50.33)	103 (52.82)	398 (47.66)	344 (53)	
somewhat less healthy	134 (7.98)	19 (9.74)	64 (7.66)	51 (7.86)	
much less healthy	72 (4.29)	10 (5.13)	28 (3.35)	34 (5.24)	
ACEs score					0.460
0	401 (23.88)	41 (21.03)	212 (25.39)	148 (22.80)	
1	510 (30.38)	66 (33.85)	249 (29.82)	195 (30.05)	
2	371 (22.10)	37 (18.97)	187 (22.40)	147 (22.65)	
3	212 (12.63)	23 (11.79)	107 (12.81)	82 (12.63)	
≥4	185 (11.02)	28 (14.36)	80 (9.58)	77 (11.86)	
ACEs indicators					
physical abuse					0.374
none	1203 (71.65)	148 (75.9)	593 (71.02)	462 (71.19)	
yes	476 (28.35)	47 (24.1)	242 (28.98)	187 (28.81)	
emotional neglect					0.727
none	1351 (80.46)	153 (78.46)	672 (80.48)	526 (81.05)	
yes	328 (19.54)	42 (21.54)	163 (19.52)	123 (18.95)	
domestic violence					0.399
none	1546 (92.08)	184 (94.36)	769 (92.1)	593 (91.37)	
yes	133 (7.92)	11 (5.64)	66 (7.9)	56 (8.63)	
peer bullying					0.964
none	1440 (85.77)	166 (85.13)	717 (85.87)	557 (85.82)	
yes	239 (14.23)	29 (14.87)	118 (14.13)	92 (14.18)	
unsafe neighborhood					0.011
none	1548 (92.2)	178 (91.28)	786 (94.13)	584 (89.98)	
yes	131 (7.80)	17 (8.72)	49 (5.87)	65 (10.02)	
parental death					0.130
none	1437 (85.59)	165 (84.62)	729 (87.31)	543 (83.67)	
yes	242 (14.41)	30 (15.38)	106 (12.69)	106 (16.33)	
parental disability (sick on bed )					0.300
none	1377 (82.01)	153 (78.46)	694 (83.11)	530 (81.66)	
yes	302 (17.99)	42 (21.54)	141 (16.89)	119 (18.34)	
parental disability (serious deformity)					0.765
none	1599 (95.24)	186 (95.38)	798 (95.57)	615 (94.76)	
yes	80 (4.76)	9 (4.62)	37 (4.43)	34 (5.24)	
sibling death					0.016
none	1379 (82.13)	172 (88.21)	667 (79.88)	540 (83.2)	
yes	300 (17.87)	23 (11.79)	168 (20.12)	109 (16.80)	
household mental illness (abnormality of mind^#^)					0.008
none	1615 (96.19)	180 (92.31)	810 (97.01)	625 (96.3)	
yes	64 (3.81)	15 (7.69)	25 (2.99)	24 (3.70)	
household mental illness (sadness or depression)					0.003
none	1337 (79.63)	142 (72.82)	690 (82.63)	505 (77.81)	
yes	342 (20.37)	53 (27.18)	145 (17.37)	144 (22.19)	
substance abuse					0.332
none	1536 (91.48)	178 (91.28)	772 (92.46)	586 (90.29)	
yes	143 (8.52)	17 (8.72)	63 (7.54)	63 (9.71)	
parental separation or divorce					0.191
none	1669 (99.4)	192 (98.46)	831 (99.52)	646 (99.54)	
yes	10 (0.60)	3 (1.54)	4 (0.48)	3 (0.46)	
incarcerated household member					0.937
none	1670 (99.46)	194 (99.49)	830 (99.4)	646 (99.54)	
yes	9 (0.54)	1 (0.51)	5 (0.60)	3 (0.46)	

ACEs, adverse childhood experiences.

a Data are presented as counts (percentage) unless otherwise indicated.

*The p-values are computed using a χ^2^ distribution.

^#^Abnormality of mind: an abnormal or disordered mental state.

### 3.2 Cognitive function trajectory modeling

The model’s optimal number of trajectory groups is 3, with a corresponding BIC value of -17308.30. By considering the morphology of the trajectory curve in the graph and the BIC value, all trajectory curves were found to be of 1st order (linear). The parameter estimates associated with the cognitive function trajectory curve can be found in **Supplementary Table 4** in the **Supplementary Material**. The model evaluation of the fitted cognitive function trajectory is presented in **Table 2**, indicating that all AvePP were more significant than 0.7, indicating a good model fit.

**Table 2:** Fit statistics of trajectory analysis.

Fit statistics	Number of class			
	2	3	4	3
Order	(2 2)	(2 2 2)	(2 2 2 2)	(1 1 1)
BIC(N=6716)	-17549.90	-17321.36	-17271.61	-17308.30
BIC(N=1679)	-17544.35	-17313.05	-17260.52	-17302.06
AIC	-17522.65	-17280.49	-17217.12	-17277.64
Class proportion, %				
Class 1	30.91	12.01	4.92	12.01
Class 2	69.10	49.44	39.6	49.44
Class 3		38.55	19.62	38.55
Class 4			35.86	
Average posterior probabilities				
Class 1	0.92	0.89	0.85	0.90
Class 2	0.96	0.92	0.80	0.92
Class 3		0.86	0.85	0.86
Class 4			0.87	

BIC, Bayesian information criterion; AIC, Akaike’s information criterion; AvePP, Average posterior probabilities.

**Figure 2:**
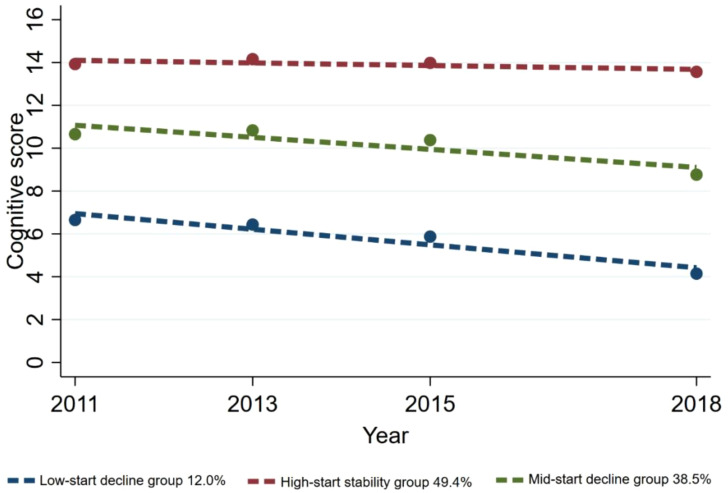
Trajectories of the cognitive function.

Ultimately, three distinct cognitive function trajectories were identified, unveiling the subsequent findings: the low-start decline group (n=195, 12.0%) exhibited an initial cognitive score at a low level and displayed a continual decline over time; the high-start stability group (n=835, 49.4%) was inclined towards higher initial cognitive scores, demonstrating minimal variability and overall stability among the study participants; the mid-start decline group (n=649, 38.5%) displayed a moderate initial cognitive score level and showed a gradual decline (**Figure 2**).

### 3.3 Relationship between ACEs and cognitive function trajectories

#### 3.3.1 Impact of baseline characteristics on cognitive function trajectories

Compared to the other two groups, the predictors of cognitive trajectory within the high-start stability group included non-agricultural Hukou, acceptance levels up to middle school, and high school/vocational school education, with corresponding odds ratios (ORs) of 6.80 (3.36-13.77), 15.58 (7.57-32.08), and 350.34 (42.9-2860.84), respectively.

Furthermore, when using the low-start decline group as a reference, participants aged 65 years or older with no childhood playmates were less inclined to be categorized as both the high-start stability group and the mid-start decline group, with OR values <1(*p* < 0.05). The low-start decline group may have an older age structure and less favorable childhood friendships (**Table 3**).

**Table 3:** Baseline factors associated with the cognitive function trajectory.

Variable	High-start stability group VS Low-start decline group		Mid-start decline group VS Low-start decline group	
	OR(95%CI)	*P* Value	OR(95%CI)	*P* Value
Gender				
male	(Reference group)			
female	0.34 (0.20-0.55)	<0.001	0.68 (0.42-1.10)	0.12
Age				
45-54	(Reference group)			
55-64	0.59 (0.37-0.95)	0.029	0.69 (0.43-1.1)	0.116
≥65	0.25 (0.15-0.4)	<0.001	0.40 (0.25-0.65)	<0.001
Residence				
agricultural Hukou	(Reference group)			
non-agricultural Hukou	6.8 (3.36-13.77)	<0.001	2.95 (1.45-6.02)	0.003
Social activity				
none	(Reference group)			
yes	1.22 (0.85-1.74)	0.277	1.17 (0.83-1.65)	0.382
Alcohol usage				
none of these	(Reference group)			
drink more than once a month	1.06 (0.66-1.69)	0.823	1.02 (0.65-1.62)	0.919
drink but less than once a month	0.73 (0.38-1.39)	0.333	0.71 (0.38-1.33)	0.287
Tobacco usage				
none	(Reference group)			
yes	0.59 (0.37-0.95)	0.03	1.01 (0.64-1.60)	0.963
Education				
no formal education	(Reference group)			
≤middle school	15.58 (7.57-32.08)	<0.001	5.46 (3.29-9.06)	<0.001
≥high school/Vocational school	350.34 (42.90-2860.84)	<0.001	31.89 (4.08-249.49)	0.001
Childhood family financial situation				
financially advantaged	(Reference group)			
financially better off	3.45 (0.5-23.72)	0.208	3.83 (0.55-26.84)	0.177
financially equal	2.00 (0.33-12.11)	0.452	2.73 (0.44-16.98)	0.281
financially somewhat worse off	1.33 (0.21-8.37)	0.762	1.55 (0.24-10.00)	0.645
financially disadvantaged	1.80 (0.29-11.25)	0.528	2.52 (0.4-16.06)	0.328
Childhood friendship				
often	(Reference group)			
sometimes	0.54 (0.33-0.89)	0.014	0.55 (0.34-0.89)	0.014
not very often	0.54 (0.30-0.99)	0.046	0.85 (0.49-1.48)	0.559
never	0.41 (0.24-0.69)	0.001	0.57 (0.35-0.92)	0.022
Earlier migration				
no move-out residence	(Reference group)			
move-out residence	1.43 (0.96-2.14)	0.08	1.17 (0.79-1.73)	0.444
Neighborhood environments				
very close-knit	(Reference group)			
somewhat close-knit	0.94 (0.65-1.37)	0.75	1.14 (0.8-1.65)	0.467
not very close-knit	0.46 (0.19-1.12)	0.089	0.72 (0.32-1.61)	0.426
not close-knit at all	0.46 (0.07-3.14)	0.425	0.59 (0.1-3.59)	0.564
Self-reported childhood health				
much healthier	(Reference group)			
somewhat healthier	0.89 (0.48-1.66)	0.719	0.59 (0.32-1.08)	0.089
about average	0.76 (0.44-1.32)	0.331	0.76 (0.45-1.30)	0.319
somewhat less healthy	0.48 (0.23-1.04)	0.063	0.51 (0.25-1.07)	0.076
much less healthy	0.61 (0.23-1.60)	0.316	0.83 (0.34-2.03)	0.679

OR, odds ratio; CI, confidence intervals.

#### 3.3.2 The relationship between cumulative ACEs and cognitive function trajectory

**Figure 3:**
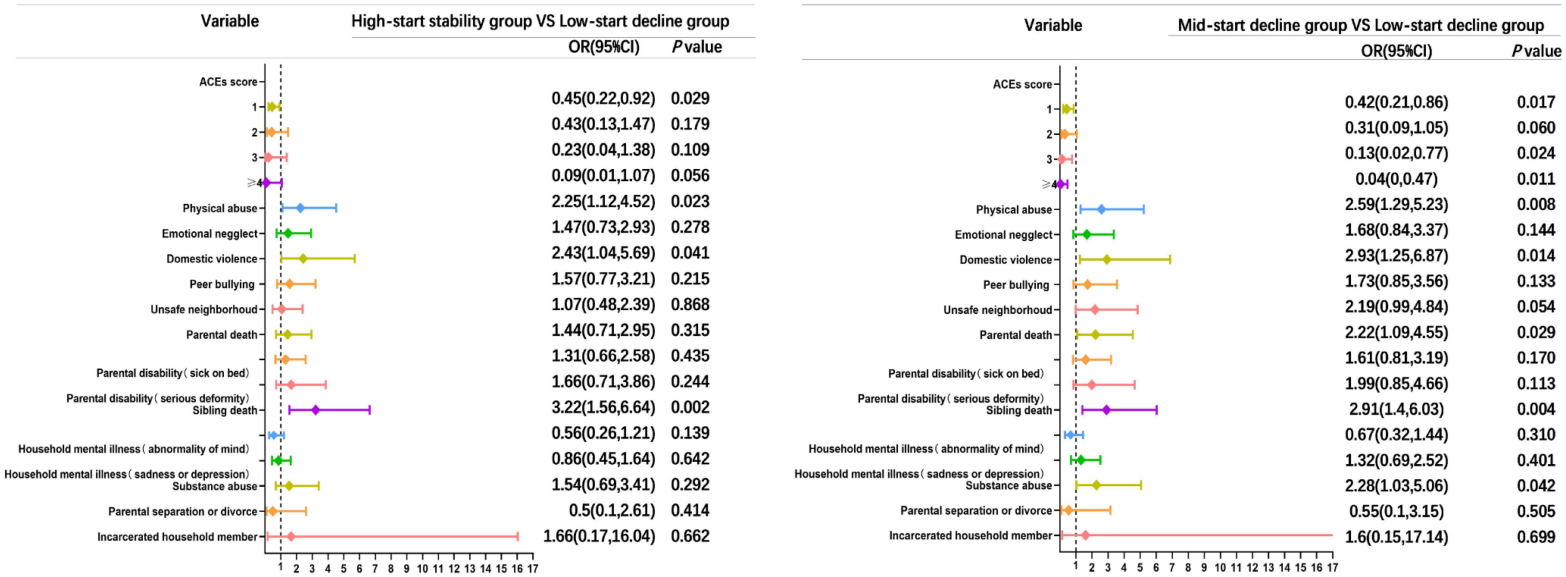
Comparison of multivariate unordered logistic forest plots of ACEs and cognitive functioning trajectories in the high-start stability group vs. low-start decline group and the mid-start decline group vs. low-start decline group. ACEs score is referenced to 0, and other entries are referenced to none. OR, odds ratio; CI, confidence intervals; ACEs, adverse childhood experiences.

As depicted in **Figure 3**, the analysis revealed no apparent dose-response correlation among accumulated ACEs with cognitive performance trajectories. However, when compared to the low-start decline group, participants who reported a single ACE, three ACEs, and four or more ACEs demonstrated a decreased likelihood of experiencing a mid-start decline trajectory, with corresponding odds ratios (ORs) of 0.42 (95% CI: 0.21-0.86), 0.13 (95% CI: 0.02-0.77), and 0.04 (95% CI: 0-0.47), respectively.

#### 3.3.3 The relationship between entry-specific ACEs and cognitive function trajectory

As depicted in **Figure 3**, when considering the high-start stability group as the focal point and utilizing the low-start decline group as the reference, the predictors associated with a cognitive trajectory for the high-start stability group included sibling death, with an odds ratio of 3.22 (95% CI: 1.56-6.64). For the mid-start decline group, the predictors of cognitive trajectory were physical abuse, domestic violence, and parental death, yielding odds ratios of 2.59 (95% CI: 1.29-5.23), 2.93 (95% CI: 1.25-6.87), and 2.22 (95% CI: 1.09-4.55), respectively.

## 4 Discussion

We concluded with three main findings. Firstly, we discovered three cognitive trajectories: low-start decline, high-start stability, and mid-start decline. Secondly, ACEs and the decline in cognitive function do not show a dose-response relationship. Thirdly, we found that the high-start stability group had specific residence and education traits, while sibling death affected them. The mid-start decline group was vulnerable to parental death, physical abuse, and domestic violence. The low-start decline group should consider age structure and childhood friendships.

Similar to previous findings, we verified a tendency toward a stable, rapid, or gradual decline in cognitive function (18, 19, 33, 34). Interestingly, the highest percentage of participants (49.4%) in this study exhibited a trajectory characterized by high-start stability in their cognitive function, suggesting that most participants maintained a stable cognitive profile over time, while the low-start decline trajectory was relatively low among middle-aged and older adults (12%). To analyze the underlying factors contributing to this phenomenon, we draw upon the theory of selective optimization with compensation (SOC) proposed by Baltes et al. (35–37). According to this theory, individuals in the high-start stability group demonstrated advantages in life experiences, such as residence and education, which contribute to the development of cognitive reserve, enabling individuals to optimize memory-related brain regions and networks. Furthermore, individuals with higher levels of cognitive reserve exhibit enhanced neuro-compensation processes, facilitating the maintenance of cognitive abilities. Conversely, the low-start decline group displayed lower baseline age and fewer childhood friendships than their counterparts. According to the disadvantage accumulation theory, weak or insufficient companies during this developmental stage may contribute to the initial vulnerability in cognitive function observed within the low-start decline group (25, 38), and these early life experiences can create barriers to overall well-being, reducing cognitive performance and health (39, 40). Thus, while early life experiences are inherent and unalterable for middle-aged and older individuals, they can serve as a basis for improving cognitive functioning.

We confirmed that there is no dose-response relationship between accumulated ACEs and cognitive function (14, 16), which may be due to the lack of validity of our subgroup analysis, where most study participants had one or fewer ACEs by the age of 17 years. Although CHARLS independently investigated the early life course of study participants, which reduced measurement bias, the ACEs scores were based on a retrospective survey, potentially underestimating the prevalence of ACEs.

We highlight the impact of early sibling and parental death experiences on cognitive abilities in the high-start stability and mid-start decline groups. Experiences of sibling and parental death early in life expose children to profound grief, which can significantly impact cognitive development. Understanding the relationship between familial death and cognition requires considering several perspectives. Firstly, multiple studies have shown that parental and sibling mortality dramatically influences cognitive functioning. Such events induce significant psychological stress, disrupting hypothalamic-pituitary-adrenal (HPA) axis regulation and increasing glucocorticoid levels. Consequently, this hormonal imbalance can shrink mature neurons in the hippocampus and reduce new neurons and precursor cells, ultimately affecting cognition (41). Secondly, the ORs of participants who had experienced sibling death were higher in both the high-start stability group and the mid-start decline group compared to the low-start decline group than those who had experienced parental death. According to death acquisition theory (41), children’s awareness of death affects cognitive development more profoundly with age, pending further testing to see if this is related to the later experience of sibling death among participants included in the study. Therefore, addressing family deaths should consider the child’s age and implement measures to prevent immediate and long-term cognitive problems associated with potential grief.

In addition, we revealed that participants who experienced physical abuse and domestic violence exhibited suboptimal cognitive functioning and a mid-start decline trajectory, and there are many physiological explanations for this phenomenon; for instance, physical abuse can result in changes in the brain, especially in the prefrontal cortex, causing ongoing psychological suffering. Similarly, exposure to domestic violence has significantly impacted the thickness and volume of cortical gray matter, potentially leading to detrimental cognitive repercussions (42–45). However, this correlation can be attributed to the higher proportion of participants in this group who have experienced physical abuse and domestic violence. This occurrence may not be coincidental in Chinese families, where strict discipline is often necessary for moral development and social harmony when children misbehave (46). These findings serve as a reminder that reducing or buffering the effects of physical abuse and domestic violence during childhood improves the immediate well-being of individuals and has long-lasting benefits for maintaining healthy cognitive functioning throughout life.

### 4.1 Strengths and limitations

We have multiple advantages. Firstly, to our knowledge, this is the first study to use group-based trajectories to explore the relationship between ACEs and cognitive functioning, incorporating up to four assessments of cognitive function, yielding relatively reliable trajectory classifications. Secondly, using directed acyclic graphs allowed us to explore genuine causal associations after adjusting for many widely recognized confounders. Thirdly, we present forest plots of cognitive subgroup predictors, which help demonstrate the relationships and influences between factors, and this combined use of statistics and data visualization makes complex data more intuitively understandable.

There are several drawbacks. Firstly, the CHARLS did not cover other sorts of ACEs, such as sexual abuse. However, sexual abuse has been associated with other ACEs and broadly correlated with cognitive function in different countries. Secondly, we adopted the method of excluding missing values during data processing. Although this method may introduce selective bias to some extent, we consider it a reasonable approach given the integrity of the study data.

### 4.2 Future research

We recommend developing potential intervention strategies based on the established cognitive trajectory in future research. Additionally, using a prospective cohort design to investigate the factors influencing the impact of ACEs on cognitive function is advisable. Further studies can focus on elucidating the potential mechanisms linking specific ACEs (such as sibling and parental death, physical abuse, and domestic violence) to cognitive decline, which may involve neurobiological research to enhance our understanding of the associated pathophysiological processes. Notably, future research should also elucidate the underlying mechanisms of positive factors that improve cognitive function to optimize early cognitive function.

### 4.3 Relevance to clinical practice

We can identify potential risk factors for cognitive decline early on based on patients’ ACEs, offering targeted intervention measures. Furthermore, given the established impact of early-life experiences on cognitive function, positive environments for children within families and society should be advocated to optimize cognitive function. These findings can serve as a foundation for developing trauma-informed care plans and public health programs to enhance cognitive function throughout the lifespan.

## 5 Conclusions

We identified trajectories of cognitive decline in middle-aged and older adults into three groups: The low-start decline group, the high-start stability group, and the mid-start decline group. We found no dose-response relationship between cognitive trajectory subgroups and cumulative ACEs. We suggest that the strengths of early life experiences (e.g., residence, education, etc.) may underlie optimized cognitive functioning. When death occurs within the family, mitigating ‘forgotten grief’ needs to consider the child’s age, as experiencing the death of siblings later in life may significantly impact cognition. Reducing physical abuse and violence in the family environment has long-term benefits for an individual’s cognitive performance.

## Data availability statement

The datasets presented in this study can be found in online repositories. The names of the repository/repositories and accession number(s) can be found below: The data supporting this study’s findings are openly available at http://charls.pku.edu.cn.

## Ethics statement

The studies involving humans were approved by The Ethical Committees of Peking University. The studies were conducted in accordance with the local legislation and institutional requirements. The participants provided their written informed consent to participate in this study.

## Author contributions

XH: Conceptualization, Data curation, Formal analysis, Methodology, Resources, Software, Supervision, Validation, Visualization, Writing – original draft, Writing – review & editing. HY: Conceptualization, Resources, Supervision, Validation, Visualization, Writing – review & editing.
