# Supplementary material for: Longitudinal association of adverse childhood experiences with cognitive function trajectories among middle-aged and older adults: group-based trajectory modeling
Source: Front Psychiatry. 2024 Aug 6;15:1440265. doi: 10.3389/fpsyt.2024.1440265 (PMC11333235; doi:10.3389/fpsyt.2024.1440265)
Supplement: Supplementary file 1 [file DataSheet_1.docx]

Supplementary Material

# Supplementary Figures and Tables

## Supplementary Figures


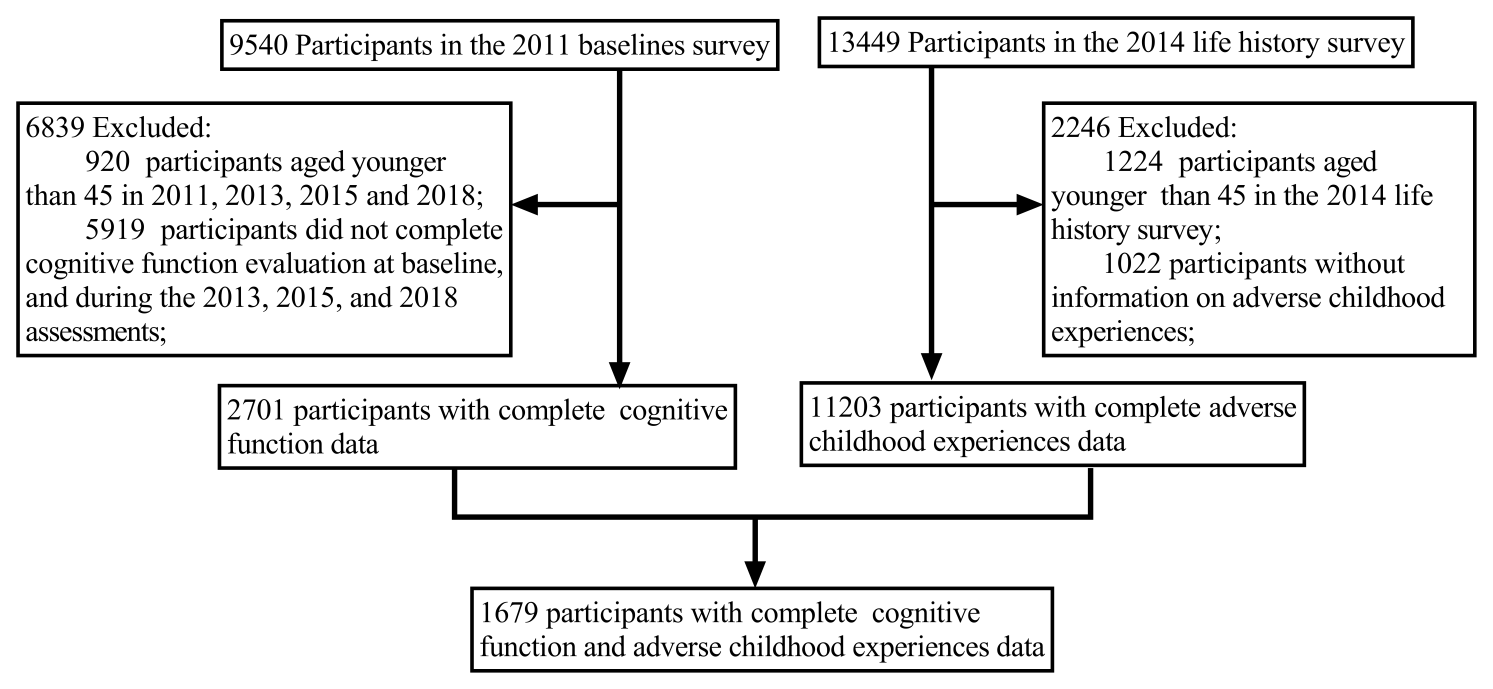


**Supplementary Figure 1.** Flowchart of the participants enrollment.

## Supplementary tables

**Supplementary table 1.** Details of specific measures of ACEs indicators items in the study.

| **ACEs categories** | **Indicators** | **Measure (Questionnaire)** | **Answer** |
| --- | --- | --- | --- |
| Child maltreatment | Physical abuse* | When you were growing up, did your female/male guardian ever hit you? | Often^#^, sometimes^#^, rarely or never |
|  | Emotional neglect* | How much love and affection did your female guardian give you while you were growing up? | Often, sometimes, rarely^#^ or never^#^ |
| Exposure to violence | Domestic violence* | How much effort did your female guardian put into watching over you? | A lot, some, a little^#^, or not at all^#^ |
|  |  | Have your father/mother ever beat up your mother/father? | Often^#^, sometimes^#^, not very often, or never |
|  | Peer bullying | When you were a child, how often were you picked on or bullied by kids in your neighborhood/ school? | Often^#^, sometimes^#^, not very often, or never |
|  | Unsafe neighborhood | Was it safe being out alone at night in the neighborhood where you lived as a child? | Very safe, somewhat safe, not very safe^#^, or not safe at all^#^ |
| Parent/sibling death or disability | Parental death ^a^ | Either of the parents was dead before you were 17 years? | (Yes^#^ or No) |
|  | Parental disability | Did your female/male guardian have a long time being sick on bed when you were young? | (Yes^#^ or No) |
|  |  | Did your female/male guardian have a serious deformity when you were young? | (Yes^#^ or No) |
|  | Sibling death ^a^ | Any of the siblings was dead before you were 17 years? | (Yes^#^ or No) |
| Parental maladjustment | Household mental illness* | Did your female/male guardian have abnormality of mind when you were young? | (Yes^#^ or No) |
|  |  | During the years you were growing up, had your female/male guardian often showed continued signs of sadness or depression? | (During all# , most# , some, or only a little of the childhood) |
|  | Substance abuse* | During the years you were growing up, did your female/male guardian ever have alcoholism or drug? | (Yes^#^ or No) |
|  | Parental separation or divorce* | Were your biological parents divorced (including long separation due to emotional problems) before you were 17 years? | (Yes^#^ or No) |
|  | Incarcerated household member* | During the years you were growing up, have your female/male guardian ever been arrested or sent to prison? | (Yes^#^ or No) |

Abbreviation: ACEs: adverse childhood experiences.

* indicated the conventional ACE indicators.

^a^ indicated that calculation is based on the dates of birth and their parents' or siblings’ death.

^#^ indicated the thresholds for ACEs_._

**Supplementary table 2.** Items adopted for the neuropsychological tests for China Health and Retirement Longitudinal Study Harmonized Cognitive Assessment Protocol(CHARLS-HCAP).

| **Cognitive Function** | **Items** | **Scores** | **Total Scores** |
| --- | --- | --- | --- |
| Episodic Memory* | Immediate word recall: by asking participants to repeat after reading ten Chinese nouns in any order immediately | 10 | 0-10 |
|  | Delayed word recall: by asking participants to repeat after reading ten Chinese nouns in any order four minutes later | 10 |  |
| Mental Status | Orientation: by asking participants to name the day, month, year, season, and correct day of the week | 5 | 0-11 |
|  | Visuoconstruction: by asking participants to re-draw a previously shown picture accurately | 1 |  |
|  | Mathematical performance: by asking participants to subtract 7 from 100 up to 5 times | 5 |  |

*Episodic Memory scores were calculated as the mean of immediate and delayed recall scores.

**Supplementary table 3.** STROBE Statement—Checklist of items that should be included in reports of cross-sectional studies.

|  | Item No | Recommendation | Page |
| --- | --- | --- | --- |
| Title and abstract | 1 | (a) Indicate the study’s design with a commonly used term in the title or the abstract | 1 |
|  |  | (b) Provide in the abstract an informative and balanced summary of what was done and what was found | 1 |
| Introduction | | | |
| Background/rationale | 2 | Explain the scientific background and rationale for the investigation being reported | 2 |
| Objectives | 3 | State specific objectives, including any prespecified hypotheses | 2 |
| Methods | | | |
| Study design | 4 | Present key elements of study design early in the paper | 2 |
| Setting | 5 | Describe the setting, locations, and relevant dates, including periods of recruitment, exposure, follow-up, and data collection | 2 |
| Participants | 6 | (a) Give the eligibility criteria, and the sources and methods of selection of participants | 2 |
| Variables | 7 | Clearly define all outcomes, exposures, predictors, potential confounders, and effect modifiers. Give diagnostic criteria, if applicable | 2-3 |
| Data sources/ measurement | 8* | For each variable of interest, give sources of data and details of methods of assessment (measurement). Describe comparability of assessment methods if there is more than one group | 2-3 |
| Bias | 9 | Describe any efforts to address potential sources of bias | NA |
| Study size | 10 | Explain how the study size was arrived at | 3 |
| Quantitative variables | 11 | Explain how quantitative variables were handled in the analyses. If applicable, describe which groupings were chosen and why | NA |
| Statistical methods | 12 | (a) Describe all statistical methods, including those used to control for confounding | 3 |
|  |  | (b) Describe any methods used to examine subgroups and interactions | NA |
|  |  | (c) Explain how missing data were addressed | 3 |
|  |  | (d) If applicable, describe analytical methods taking account of sampling strategy | NA |
|  |  | (e) Describe any sensitivity analyses | 10 |
| Results | | | |
| Participants | 13* | (a) Report numbers of individuals at each stage of study—eg numbers potentially eligible, examined for eligibility, confirmed eligible, included in the study, completing follow-up, and analysed | 3 |
|  |  | (b) Give reasons for non-participation at each stage | NA |
|  |  | (c) Consider use of a flow diagram | Figure 1 in the supplementary material |
| Descriptive data | 14* | (a) Give characteristics of study participants (eg demographic, clinical, social) and information on exposures and potential confounders | Table 1 |
|  |  | (b) Indicate number of participants with missing data for each variable of interest | 2 |
| Outcome data | 15* | Report numbers of outcome events or summary measures | NA |
| Main results | 16 | (a) Give unadjusted estimates and, if applicable, confounder-adjusted estimates and their precision (eg, 95% confidence interval). Make clear which confounders were adjusted for and why they were included | NA |
|  |  | (b) Report category boundaries when continuous variables were categorized | Table 1 |
|  |  | (c) If relevant, consider translating estimates of relative risk into absolute risk for a meaningful time period | NA |
| Other analyses | 17 | Report other analyses done—eg analyses of subgroups and interactions, and sensitivity analyses | NA |
| Discussion | | | |
| Key results | 18 | Summarise key results with reference to study objectives | 3 |
| Limitations | 19 | Discuss limitations of the study, taking into account sources of potential bias or imprecision. Discuss both direction and magnitude of any potential bias | 10 |
| Interpretation | 20 | Give a cautious overall interpretation of results considering objectives, limitations, multiplicity of analyses, results from similar studies, and other relevant evidence | 10-11 |
| Generalisability | 21 | Discuss the generalisability (external validity) of the study results | NA |
| Other information | | | |
| Funding | 22 | Give the source of funding and the role of the funders for the present study and, if applicable, for the original study on which the present article is based | NA |

*Give information separately for exposed and unexposed groups.

Note: An Explanation and Elaboration article discusses each checklist item and gives methodological background and published examples of transparent reporting. The STROBE checklist is best used in conjunction with this article (freely available on the Web sites of PLoS Medicine at http://www.plosmedicine.org/, Annals of Internal Medicine at http://www.annals.org/, and Epidemiology at http://www.epidem.com/). Information on the STROBE Initiative is available at [www.strobe-statement.org.](http://www.strobe-statement.org.)

**Supplementary table 4.** Cognitive function trajectory curve-related parameters.

| Class | Characteristics of the trajectory | Parameter | Estimate | Standard Error | *t* | *p* |
| --- | --- | --- | --- | --- | --- | --- |
| 1 | Low-start decline group | Intercept | 747.78 | 83.83 | 8.92 | ＜0.01 |
|  |  | Linear | -0.37 | 0.04 | -8.85 | ＜0.01 |
| 2 | High-start stability group | Intercept | 135.32 | 40.047 | 3.38 | 0.0007 |
|  |  | Linear | -0.06 | 0.02 | -3.03 | 0.0024 |
| 3 | Mid-start decline group | Intercept | 576.32 | 49.35 | 11.68 | ＜0.01 |
|  |  | Linear | -0.28 | 0.02 | -11.47 | ＜0.01 |
